# Early Revegetation Initiates Soil Physical Habitat Reconstruction and Microbial Community Reassembly Within the First Growing Season in an Alpine Mining Substrate

**DOI:** 10.3390/plants15203173

**Published:** 2026-10-16

**Authors:** Shuhan Zhang, Bo Cai, Xiangqian Zhao, Jing Xiang, Xihui Sun, Lixin Niu, Xianzhi Wang, Yanlong Zhang

**Affiliations:** 1 College of Landscape Architecture & Arts, Northwest A&F University, Yangling 712100, China; zhangshuhan@nwafu.edu.cn (S.Z.); caibo@nwafu.edu.cn (B.C.); 2024057012@nwafu.edu.cn (X.Z.); xiangjing@nwafu.edu.cn (J.X.); sunxihui@nwafu.edu.cn (X.S.); niulixin@nwsuaf.edu.cn (L.N.); 2 China MCC5 Group Corp. Ltd., Chengdu 610063, China

**Keywords:** Qinghai–Tibet Plateau, alpine mining area, soil pore structure, soil microbial community, pioneer grass

## Abstract

Ecological restoration of alpine mining areas is constrained by low temperature, infertile substrates, and limited water availability, yet the coordinated early responses of plants, soils, and microorganisms remain poorly understood. We conducted a 90-day in situ revegetation experiment in an alpine sand-and-gravel mine in Tibet using three native perennial grasses, *Elymus nutans*, *Agropyron cristatum*, and *Festuca sinensis*, established in monoculture and mixed-sowing treatments. Plant growth and physiology, soil physicochemical properties, nutrients, enzyme activities, and microbial communities were assessed. All three pioneer species established measurable biomass within the first growing season, although growth and physiological responses differed among planting configurations. Soil capillary porosity, moisture content, and water-holding capacity showed the most consistent improvements, whereas enzyme activities and nutrient responses were more treatment-dependent. Bacterial and fungal communities underwent significant reorganization, while inferred co-occurrence networks showed temporal topological restructuring and sensitivity to simulated targeted node removal. Mantel analysis showed that bacterial community variation was significantly associated with capillary porosity, non-capillary porosity, and total phosphorus, whereas fungal community variation was not significantly associated with the measured soil variables. Overall, 90 days of pioneer revegetation initiated measurable plant–soil–microbial recovery in the alpine mining substrate. Physical and hydrological properties showed clearer early responses than most nutrient indicators, while the observed associations between soil pore characteristics and bacterial community composition suggest a potential link between physical habitat changes and microbial community reassembly.

## 1. Introduction

The Qinghai–Tibet Plateau hosts globally significant alpine ecosystems whose vulnerability is amplified by the combined constraints of high altitude (>3500 m), low mean annual temperature, and limited precipitation [1,2]. These climatic constraints can slow nutrient cycling and limit plant establishment in degraded alpine soils [3]. Against this backdrop, expanding mineral exploitation has produced extensive surface disturbances, including the loss of vegetation cover, disruption of soil structure, and depletion of nutrient pools, for which natural succession alone is often insufficient to achieve rapid recovery on management-relevant timescales [4,5,6]. Therefore, active vegetation restoration is an important strategy for halting further degradation in such alpine mining areas.

Vegetation restoration is widely accepted as a primary strategy for restoring soil ecological functions in mining areas, operating through root inputs, litter accumulation, and rhizosphere conditioning [7,8,9]. Native pioneer species are often favored in mine restoration because their adaptation to local climatic and edaphic conditions can facilitate establishment and support ecosystem recovery [10,11]. Beyond species identity, mixed sowing is widely advocated on the basis that interspecific niche differentiation and resource complementarity yield greater long-term productivity and stability than monocultures [12,13,14]. Biodiversity-ecosystem-functioning experiments consistently show that positive biodiversity effects emerge gradually, often requiring multiple growing seasons, while early productivity is often dictated by the competitive dominance of the single best-performing species [15,16]. Consequently, the relative short-term performance of monoculture and mixed-sowing configurations remains uncertain in severely disturbed alpine mining substrates.

Restoration outcomes are most often assessed through aboveground proxies such as cover or biomass, with belowground responses examined less systematically and rarely in the same study [17,18,19]. This matters, because the soil microbiome is not merely a downstream beneficiary of plant establishment: bacterial and fungal communities form interactive networks whose topology, keystone taxa, and successional trajectory shape the rate at which soil functions can recover [20,21]. Mining disturbance can strongly alter soil microbial diversity and community composition, potentially constraining the recovery of belowground ecological functions [22]. Under these conditions, the identity and connectivity of early colonizing microbes likely exert a disproportionate influence on whether vegetation establishment translates into functional recovery [23,24]. In recently disturbed substrates, microbial colonization is also constrained by the physical architecture of the soil matrix. Pore structure regulates water retention, aeration, solute diffusion, and the spatial connectivity of microbial habitats, and may therefore be particularly important during early community establishment [25,26]. Although previous studies have documented mining-induced changes in soil microbial communities and highlighted the importance of soil pore structure for microbial habitats, the relationships among vegetation establishment, substrate physical changes, and microbial community reassembly during early restoration in alpine mining areas remain insufficiently understood. In particular, whether changes in soil physical and hydrological properties are associated with bacterial and fungal community reassembly within the first growing season remains unclear.

To address these gaps, we conducted a 90-day in situ restoration experiment at an alpine sand-and-gravel mine in Tibet. We used three perennial grasses (*Elymus nutans*, *Agropyron cristatum*, and *Festuca sinensis*). Species selection was informed by field observations of *E. nutans* and members of Festuca and Agropyron in the surrounding vegetation, together with previous studies on their restoration potential. Previous studies have demonstrated the application of *E. nutans* and *F. sinensis* in alpine mining restoration and examined their physiological adaptation to low-temperature conditions on the Qinghai–Tibet Plateau [27,28]. Meanwhile, *A. cristatum* has been identified as a candidate species for revegetation in arid mining environments [29]. These considerations motivated the selection of the three species to evaluate their early establishment and restoration performance under local alpine mining conditions. The three species were arranged in three monocultures, four mixed-sowing treatments (three two-species and one three-species), and a bare-soil control, and we tracked plant growth and physiology, soil physicochemical properties, nutrients, enzyme activities, and microbial communities. We tested three hypotheses: (H1) Native pioneer grasses can establish measurable biomass and maintain physiological activity within the first growing season under alpine mining stress, although their responses may differ among planting configurations; (H2) Early vegetation establishment rapidly improves the physical and hydrological properties of the mining substrate, whereas changes in bulk nutrient status remain comparatively limited during the first growing season; and (H3) Pioneer vegetation establishment induces measurable reorganization of soil bacterial and fungal communities during the first growing season, and this early microbial reassembly is associated with changes in the substrate microenvironment, particularly soil physical structure. By testing these hypotheses jointly, we aimed to identify the plant- and microbe-level processes most tightly coupled to early substrate amelioration.

## 2. Results

### 2.1. Plant Growth Phenotypes and Physiological Indices

**Figure 1:**
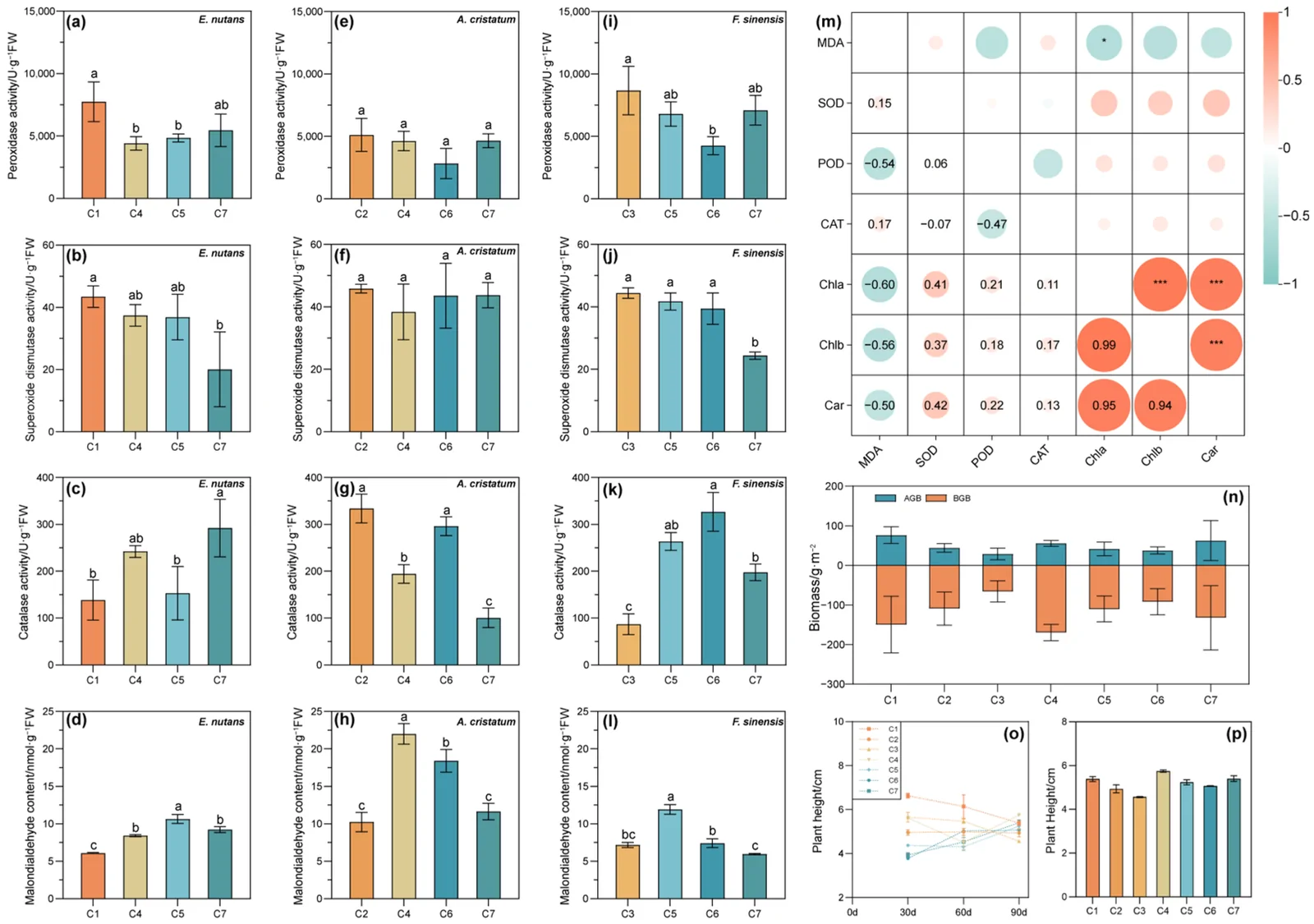
Growth and physiological responses of pioneer plants under different planting treatments. (**a**–**l**) Antioxidant enzyme activities (SOD, CAT, and POD) and MDA contents of individual plant species across different treatments. (**m**) Correlation analysis of plant physiological indicators. (**n**) Aboveground and belowground biomass after 90 days. (* *p* < 0.05 and *** *p* < 0.001). (**o**,**p**) Changes in plant height during the experiment. The letters above the bars indicate significant differences between the mean values according to Tukey’s HSD post hoc test (*p* < 0.05).

The photosynthetic pigment contents (Chl a, Chl b, and Car) in the leaves of *E. nutans* were lower in C7 than in C1, C4, and C5. For *A. cristatum*, the photosynthetic pigment contents in C2 were significantly higher than those in C4, C6, and C7 (Figure S2). Meanwhile, *F. sinensis* maintained relatively high photosynthetic pigment contents in C3 and C6. Photosynthetic pigment contents showed species-specific responses to planting configuration. In C1, the MDA content of *E. nutans* (6.10 nmol·g^−1^ FW) was significantly lower than in C4, C5, and C7. Furthermore, *E. nutans* in C1 displayed the highest leaf POD and SOD activities, alongside the lowest CAT activity (Figure 1a–d). For *A. cristatum*, the lowest MDA content was observed in C2, whereas the highest was recorded in C4 (22.0 nmol·g^−1^ FW) (Figure 1h). The leaf CAT activity of *A. cristatum* in C2 was significantly higher than in other treatments, while its POD and SOD activities showed no significant differences across treatments (Figure 1e–g). *F. sinensis* exhibited relatively low MDA contents in C3 and C7, measuring 7.17 and 5.96 nmol·g^−1^ FW, respectively. In C3, *F. sinensis* possessed the highest leaf POD and SOD activities and the lowest CAT activity (Figure 1i–l).

Pearson correlation analysis of plant physiological indicators (Figure 1m) revealed significant positive correlations among Chl a, Chl b, and Car (*p* < 0.05). MDA was negatively correlated with Chl a (*p* < 0.05), and showed non-significant negative correlations with POD, Chl b, and Car (*p* > 0.05). At day 90, the average plant height across treatments ranged from 4.6 to 5.8 cm (Figure 1o,p). C1 achieved the highest aboveground biomass (76.3 g·m^−2^), whereas C4 recorded the highest belowground biomass (169.7 g·m^−2^). C3 had the lowest aboveground and belowground biomass, at 28.7 and 65.7 g·m^−2^, respectively (Figure 1n). C1, C4, and C7 showed relatively high total biomass, ranging from 195.0 to 226.0 g·m^−2^.

### 2.2. Soil Physicochemical Properties

**Figure 2:**
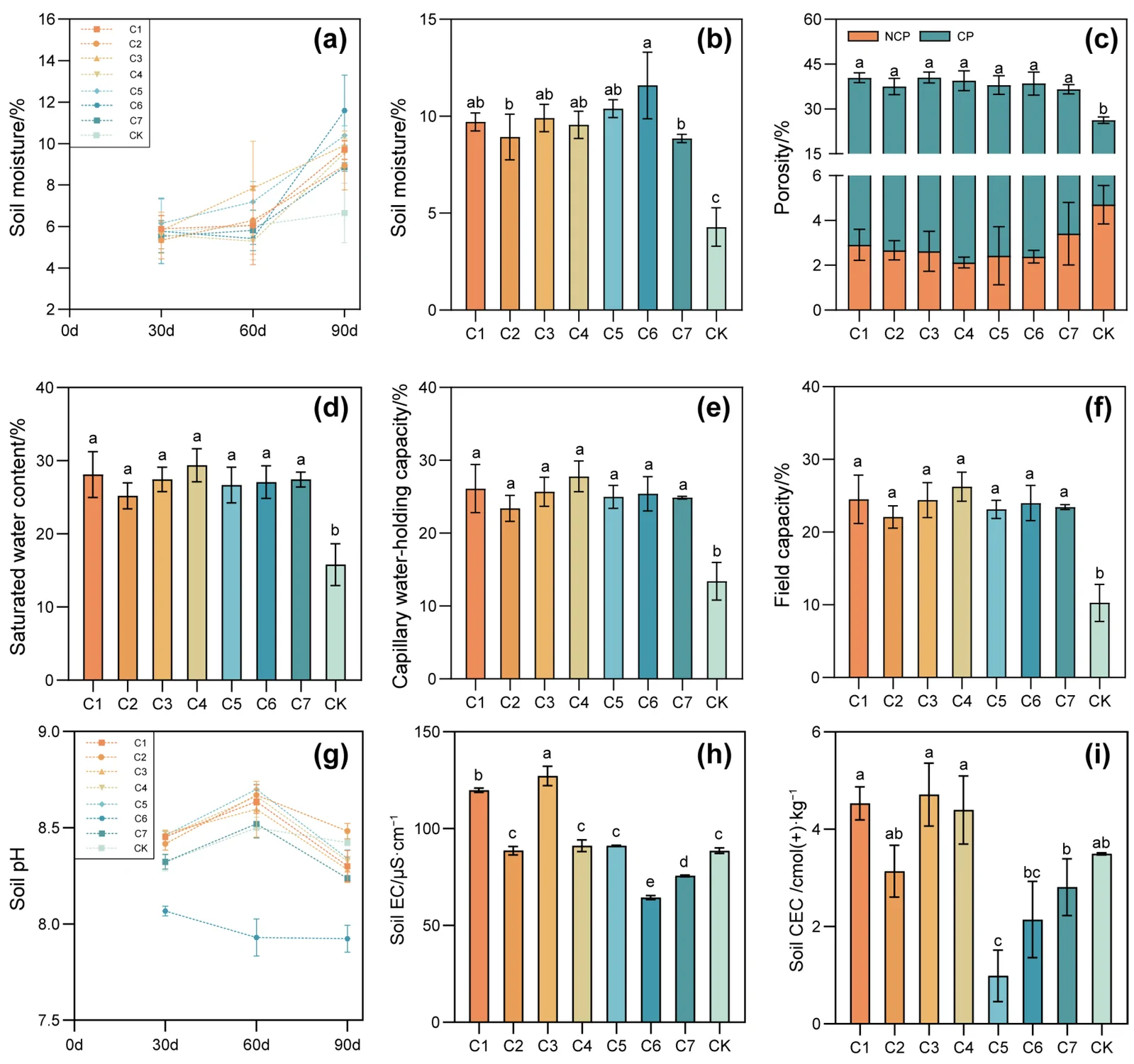
Soil physicochemical properties under different planting treatments. (**a**,**b**) Dynamics of soil moisture throughout the experiment and the final moisture content at day 90. (**c**) Soil porosity (CP and NCP). (**d**–**f**) Saturated moisture content, capillary water-holding capacity, and field capacity, respectively. (**g**–**i**) Soil chemical properties, including pH, EC, and CEC. The letters above the bars indicate significant differences between the mean values according to Tukey’s HSD post hoc test (*p* < 0.05).

At day 0, the disturbed mining substrate exhibited substantially lower soil water content (SWC; 2.61% vs. 8.91%), capillary porosity (CP; 21.76% vs. 38.12%), and cation exchange capacity (CEC; 0.97 vs. 3.45 cmol(+)·kg^−1^) than the adjacent undisturbed reference soil. In contrast, bulk density (1.64 vs. 1.45 g·cm^−3^) and pH (8.38 vs. 6.66) were higher in the disturbed substrate (Table S1). These initial characteristics indicated a relatively compact, alkaline, water-limited substrate environment before revegetation. At day 90, SWC and CP were significantly higher in the planted treatments than in CK (*p* < 0.05), whereas non-capillary porosity (NCP) was lower (Figure 2b,c). CP was 1.42–1.54 times that in CK, whereas NCP was 27.43–54.86% lower than CK. Soil bulk density was lower in all planted treatments than in CK, but significant differences were observed only in C4 and C7 (*p* < 0.05; Figure S3). Soil saturated moisture content, capillary water-holding capacity, and field capacity were significantly higher in the planted treatments than in CK (*p* < 0.05) (Figure 2d–f). The pH of CK increased from 8.32 (30 days) to 8.42 (90 days) (Figure 2g). Among the planted treatments, pH showed a decreasing trend in all treatments except C2. At day 90, electrical conductivity (EC) was significantly higher in C1 and C3 than in CK, but significantly lower in C6 and C7 (*p* < 0.05) (Figure 2h). Compared to CK (3.50 cmol(+)·kg^−1^), only C5 (0.99 cmol(+)·kg^−1^) exhibited a significant decrease in CEC (*p* < 0.05) (Figure 2i). Overall, physical and hydrological properties showed relatively consistent responses to vegetation establishment, whereas soil chemical properties exhibited more treatment-specific changes.

### 2.3. Soil Nutrients and Enzyme Activities

**Figure 3:**
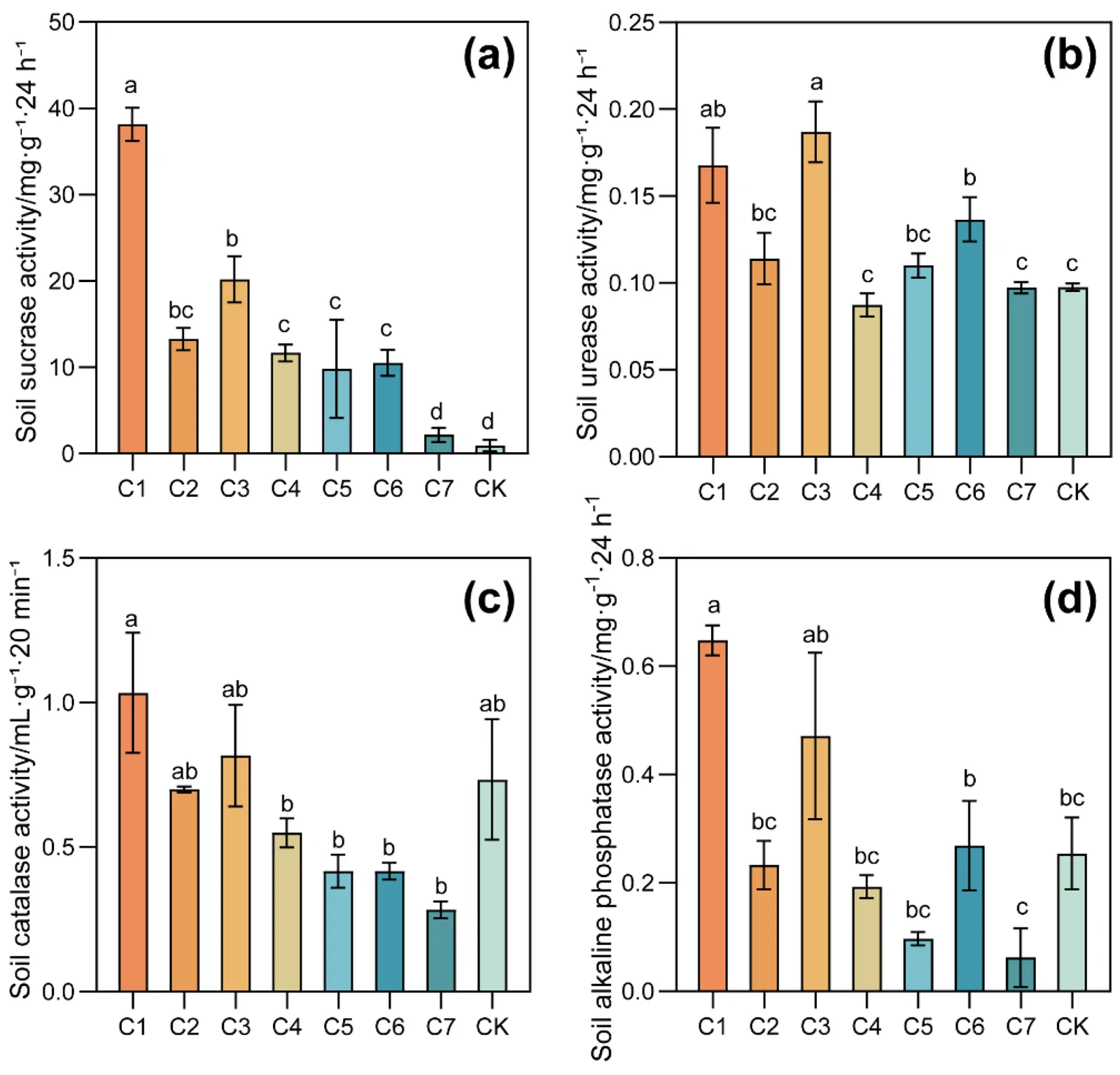
Responses of soil enzyme activities to different planting treatments. (**a**–**d**) Activities of sucrase, urease, catalase, and alkaline phosphatase in the substrate, respectively. The letters above the bars indicate significant differences between the mean values according to Tukey’s HSD post hoc test (*p* < 0.05).

All three monocultures showed higher sucrase activities (13.27–38.14 mg·g^−1^·24 h^−1^) than the mixed-sowing treatments (2.18–11.68 mg·g^−1^·24 h^−1^). CK recorded the lowest activity at 0.94 mg·g^−1^·24 h^−1^ (Figure 3a). Soil sucrase activity was highest in C1 at 38.14 mg·g^−1^·24 h^−1^, approximately 40.6 times that in CK (0.94 mg·g^−1^·24 h^−1^; *p* < 0.05). C1 had the highest alkaline phosphatase activity (0.65 mg·g^−1^·24 h^−1^) and was the only treatment significantly higher than CK (0.25 mg·g^−1^·24 h^−1^) (*p* < 0.05) (Figure 3d). Urease activity was significantly higher in C1, C3, and C6 than in CK (*p* < 0.05), with C3 reaching a peak at 0.18 mg·g^−1^·24 h^−1^ (Figure 3b). Catalase activity was relatively high in C1 and C3. In the mixed-sowing treatments, catalase activity ranged from 0.28 to 0.55 mL·g^−1^·20 min^−1^, lower than the value observed in CK (0.73 mL·g^−1^·20 min^−1^).

NH_4_^+^-N content was significantly higher than CK (0.74 mg·kg^−1^) in C1 (1.79 mg·kg^−1^) and C5 (1.09 mg·kg^−1^, Table S2). Compared to CK, AK contents in C1, C3, and C6 (75.28, 89.24, and 61.31 mg·kg^−1^, respectively) were significantly higher than in CK. NO_3_^−^-N levels were 6.39, 15.81, and 2.82 mg·kg^−1^, respectively (*p* < 0.05). No significant changes were observed in the other treatment groups. TP contents in all planted treatments (1.27–1.94 g·kg^−1^) were higher than that in CK (0.98 g·kg^−1^). However, the planted treatments exhibited no significant differences in SOC, AP and TN contents relative to CK.

### 2.4. Soil Microbial Diversity and Taxonomic Composition

**Figure 4:**
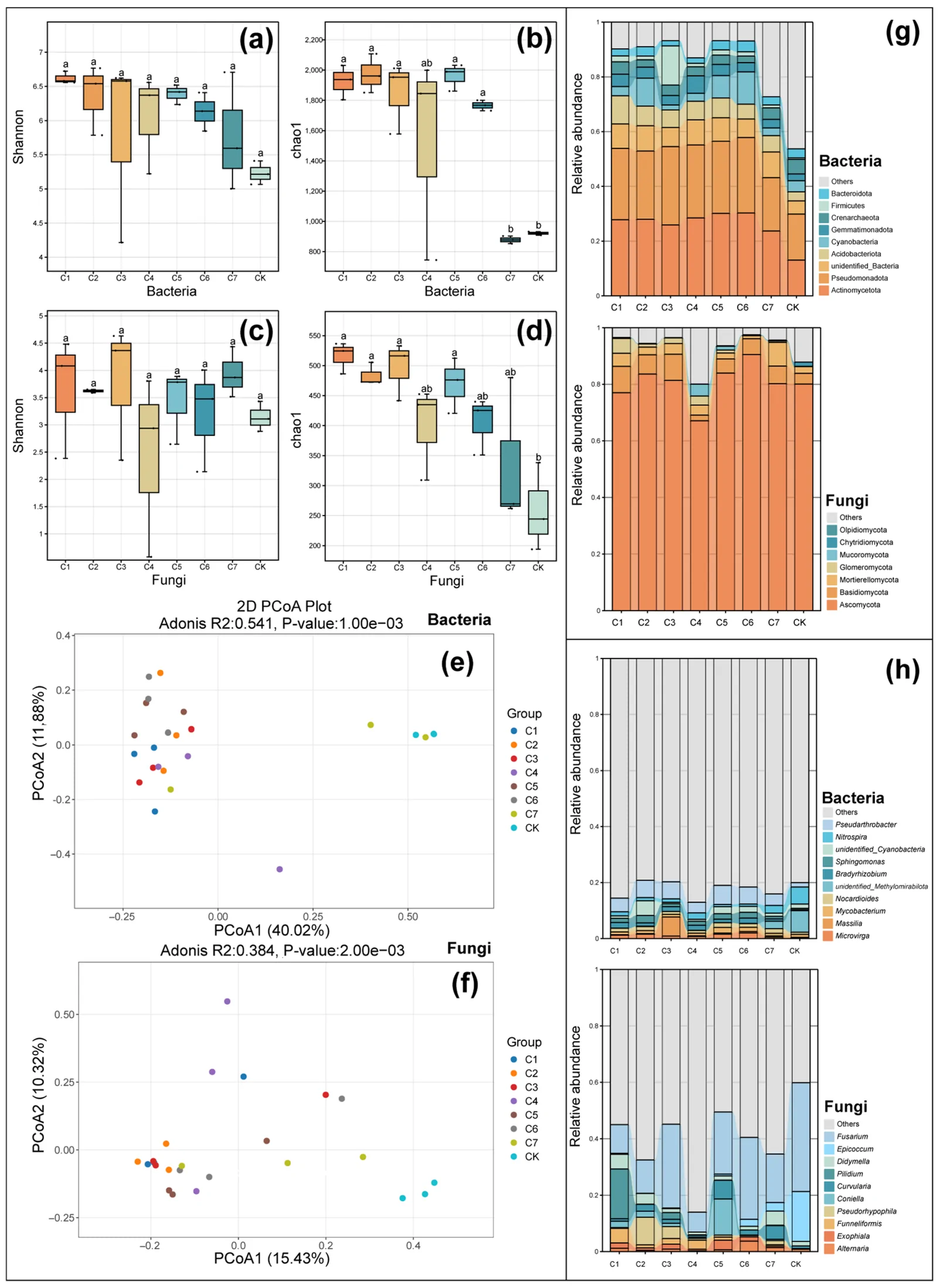
Diversity and community structure of soil bacteria (**a**,**b**,**e**) and fungi (**c**,**d**,**f**) under different treatments at day 90. Taxonomic composition of soil microbial communities at the (**g**) phylum and (**h**) genus levels across different planting treatments. Different lowercase letters indicate significant differences among treatments according to Tukey’s HSD test (*p* < 0.05).

Alpha diversity analysis (Figure 4a–d) showed that bacterial and fungal richness responded more strongly to planting treatments than Shannon diversity. For bacterial Chao1, C1, C2, C3, C5, and C6 were significantly higher than CK, whereas C4 and C7 did not differ significantly from CK (Figure 4b). For fungal Chao1, C1, C2, C3, and C5 were significantly higher than CK, while C4, C6, and C7 showed no significant differences from CK (Figure 4d; *p* > 0.05). In contrast, neither bacterial nor fungal Shannon diversity differed significantly among treatments (Figure 4a, c; *p* > 0.05). PCoA visualized treatment-associated variation in bacterial and fungal community composition (Figure 4e,f). PERMANOVA revealed significant differences among treatments for both bacteria (pseudo-*F*(7,16) = 2.692, *R*^2^ = 0.541, *p* < 0.01) and fungi (pseudo-*F*(7,16) = 1.424, *R*^2^ = 0.384, *p* < 0.01). However, PERMDISP detected significant differences in within-group multivariate dispersion for fungi (*F*(7,16) = 2.657, *p* = 0.0449), but not for bacteria (*F*(7,16) = 1.182, *p* = 0.3560). These results suggest that differences in within-group dispersion may have contributed to the significant fungal PERMANOVA result. The dominant bacterial phyla were Actinomycetota (13.0–30.3%), Pseudomonadota (16.8–28.6%), and Acidobacteriota (3.4–10.3%) (Figure 4g). Fungi were overwhelmingly composed of Ascomycota (77.0–90.5%). At the genus level, planted treatments generally showed higher relative abundances of *Exophiala*, *Funneliformis*, *Pseudorhypophila*, *Coniella*, *Pilidium*, *Microvirga*, *Nocardioides*, *Bradyrhizobium*, *Sphingomonas*, and *Pseudarthrobacter*, whereas *Fusarium*, *Epicoccum*, and *Nitrospira* tended to show lower relative abundances than in CK (Figure 4h).

### 2.5. Differentially Abundant Microbial Taxa and Temporal Co-Occurrence Network Dynamics

**Figure 5:**
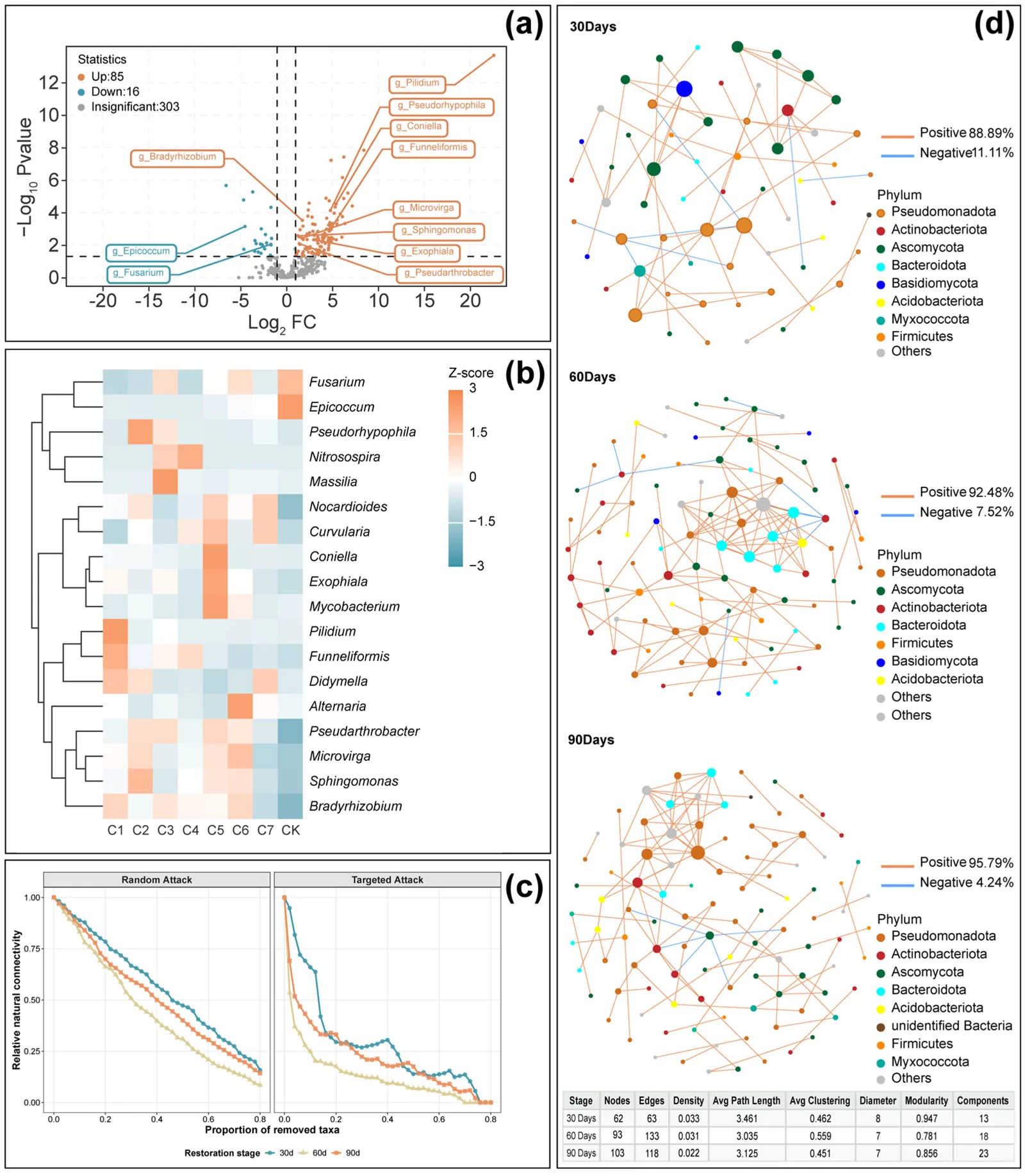
Differentially abundant microbial taxa and temporal co-occurrence network dynamics in the alpine mining substrate. (**a**) DESeq2 analysis of differentially abundant microbial genera between planted treatments and CK at day 90. (**b**) Effects of different planting treatments on the abundances of selected microbial genera in the mining substrate at day 90. (**c**) Network robustness under random and targeted attacks at 30, 60, and 90 d. (**d**) Co-occurrence networks constructed from the seven planted treatments at 30, 60, and 90 d, with node sizes proportional to their degree and selected taxa labeled.

DESeq2 analysis comparing the pooled planted group (C; C1–C7, *n* = 21) with CK (*n* = 3) at day 90 identified 101 differentially abundant genera, including 85 enriched and 16 depleted genera in the planted group relative to CK (Figure 5a). Notably, *Fusarium* was significantly depleted in C1, C2, and C4 relative to CK, decreasing by 88.18%, 83.68%, and 87.23%, respectively (Figure 5b; *p* < 0.05). Several bacterial genera containing strains previously associated with plant-beneficial traits were enriched. Specifically, *Sphingomonas* abundance was 2.53–3.16 times that in CK in C2, C5, and C6; *Bradyrhizobium* was significantly enriched 2.96–6.00 times that in CK in C1, C3, C4, and C6 (*p* < 0.05). Concurrently, *Microvirga* abundance was 2.42–4.55 times that in CK in the planting groups except C4 and C7 (*p* < 0.05). Stage-specific co-occurrence networks showed marked temporal changes during the 90-day restoration period (Figure 5d). The number of nodes increased from 62 at day 30 to 103 at day 90, while the proportion of positive correlations progressively increased from 88.89% at 30 days to 92.48% at 60 days and 95.79% at 90 days. However, the modularity index decreased from 0.947 to 0.856, and network density decreased from 0.033 to 0.022. Robustness analysis further showed that relative natural connectivity declined rapidly under targeted node removal at all three stages, with a pronounced decrease within the first approximately 20% of targeted node removal (Figure 5c).

### 2.6. Soil Environmental Factors Associated with Early Microbial Community Reorganization

**Figure 6:**
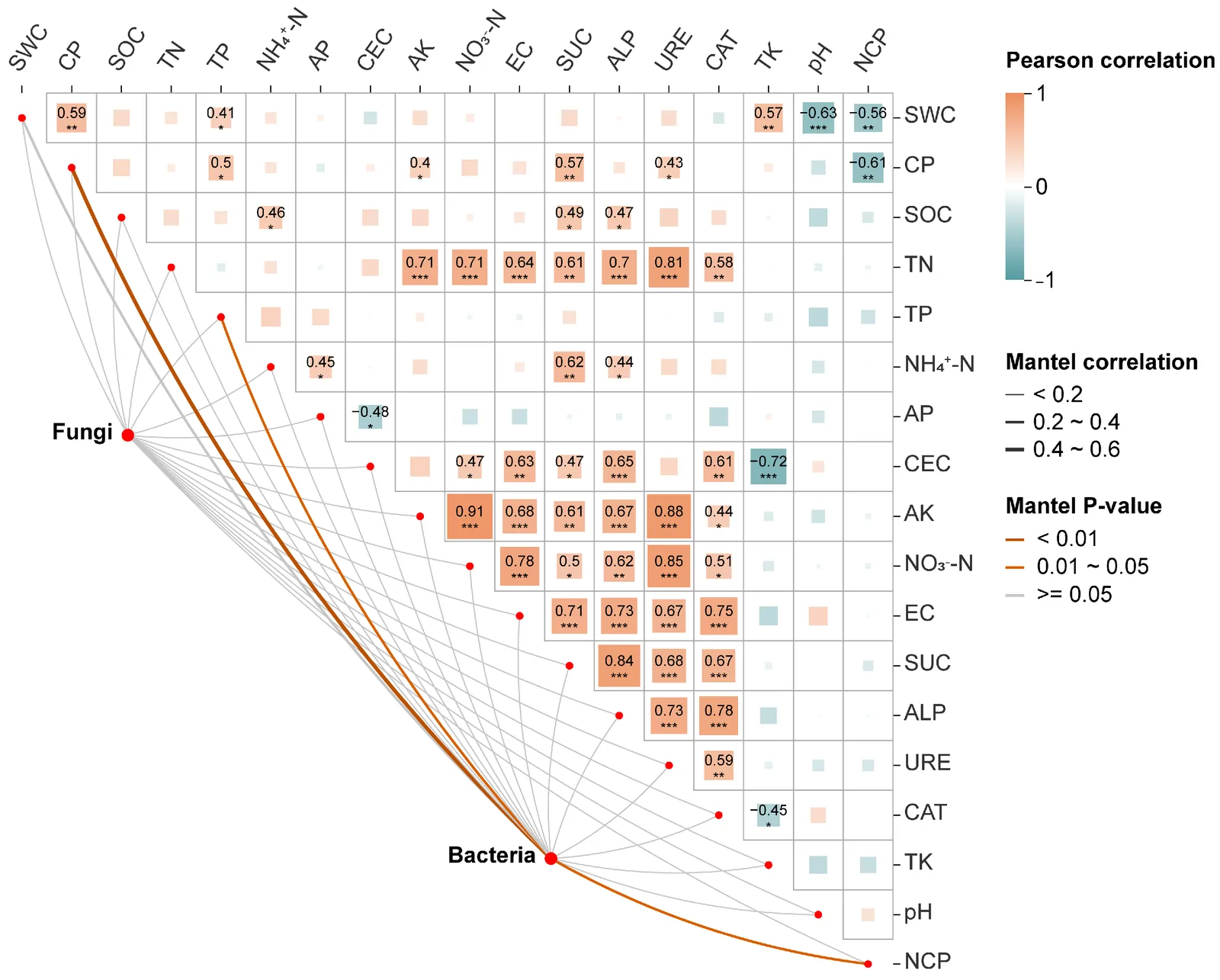
Mantel test and Pearson correlation analysis between microbial community structures and soil microhabitat indicators at day 90. Line width represents Mantel r, line color represents Mantel p, asterisks in the matrix indicate Pearson significance. (* *p* < 0.05, ** *p* < 0.01, and *** *p* < 0.001).

To identify the environmental factors associated with early microbial community reorganization during the first growing season, Mantel tests and Pearson correlation analyses were conducted between bacterial and fungal community structures and 18 soil indicators (Figure 6). Pearson correlation analysis showed significant positive associations among several soil enzyme activities (SUC, ALP, URE, and CAT), nutrient variables (TN, AK, NO_3_^−^-N), and EC. pH exhibited a highly significant negative correlation with SWC (*p* < 0.001), while NCP showed a significant negative correlation with both SWC and CP (*p* < 0.01). The Mantel test revealed contrasting relationships between soil environmental variables and bacterial versus fungal community structures. None of the measured soil variables showed a significant association with fungal community structure (*p* > 0.05). Bacterial community structure was significantly associated with CP, NCP, and TP (*p* < 0.05), whereas no significant associations were detected for the remaining variables. These results indicate that, among the measured environmental variables, bacterial community variation was most closely associated with soil pore characteristics (CP and NCP) and TP during the first growing season.

## 3. Discussion

### 3.1. Plant Community Establishment and Physiological Responses During the First Growing Season

The strong concentration of root biomass in shallow alpine soils may facilitate access to near-surface soil resources, particularly water [30]. After 90 days, the planted communities had accumulated 94.3–226.0 g·m^−2^ of total biomass (Figure 1n). Among the monocultures, *E. nutans* showed the highest aboveground biomass, indicating strong early biomass accumulation during the first growing season. In response to the harsh environment, alpine plants must activate abiotic stress defense mechanisms, using antioxidant enzymes to scavenge excess reactive oxygen species (ROS) [31,32]. MDA is widely used as an indicator of membrane lipid peroxidation and oxidative injury under abiotic stress [28]. Enzymes such as SOD, POD, and CAT are mobilized to counteract this oxidative stress [33,34]. Under environmental stresses such as drought, ROS overaccumulation typically suppresses photosynthetic capacity by disrupting chloroplast structures and accelerating pigment degradation [35]. In this study, physiological responses to planting configuration were species-specific, although relatively high pigment contents were frequently observed in monocultures. Correlation coefficients between MDA and all three pigments were negative, but only the relationship between MDA and Chl a was statistically significant (Figure 1m). The significant negative relationship between Chl a and MDA suggests that higher Chl a content was associated with lower membrane lipid peroxidation under the experimental conditions. Antioxidant enzyme activities may also vary with developmental stage, tissue condition, and metabolic activity; therefore, the observed differences alone cannot establish differential stress tolerance among treatments. Although monocultures showed relatively favorable responses for several traits, the 90-day establishment period is insufficient to infer their long-term superiority over mixed plantings.

### 3.2. Effects of Early Vegetation Restoration on the Substrate Environment in Alpine Mining Areas

Alpine herbaceous plants can develop root systems to ameliorate the physical structure of the substrate. Plant roots can substantially reorganize soil pore architecture through biopore formation, root expansion, and displacement of soil particles, although the direction and magnitude of these effects depend on the initial soil structure [36,37]. After 90 days, CP reached 37.53–40.53% in the planted treatments, compared with 26.25% in CK, while SWC reached 8.86–11.59%, compared with 4.28% in CK (Figure 2b,c). The concurrent increases in saturated moisture content, capillary water-holding capacity, and field capacity further indicate that vegetation establishment rapidly altered the hydrological functioning of the mining substrate (Figure 2d–f). The initial characterization revealed substantially lower SWC and CP, together with higher bulk density, in the disturbed mining substrate than in the adjacent undisturbed reference soil (Table S1). Although CP in the planted treatments after 90 days was numerically comparable to the initial reference-soil values, this does not indicate complete soil recovery, given the differences in sampling locations and times. Rather, the comparisons with CK provide evidence of early, partial improvements in the physical and hydrological properties of the mining substrate. These changes in soil porosity, bulk density, and water-holding properties provide evidence of early physical habitat modification. However, soil aggregate stability and detailed pore-size distribution were not directly measured; therefore, the extent of soil structural reconstruction cannot be fully determined from the present data.

During early establishment, abiotic stress can alter the release and composition of root exudates, including sugars and organic acids, which may contribute to resource acquisition and plant–microbe interactions [38,39,40]. Soil enzyme responses were rapid but treatment-dependent. Sucrase activity responded strongly to vegetation establishment, particularly in monocultures, whereas significantly higher urease activity was restricted to C1, C3, and C6 (Figure 3a,b). In contrast, alkaline phosphatase and catalase showed more selective or inconsistent responses (Figure 3c,d). Sucrase and urease are commonly associated with soil C turnover and N mineralization, respectively. Their elevated activities in several treatments suggest an early response of substrate biochemical processes [41], and are consistent with increased microbial nutrient turnover [41,42]. Bulk nutrient concentrations showed comparatively limited and treatment-specific differences relative to CK. SOC and TN did not differ significantly from CK, whereas TP differed from CK in most planted treatments and TK differed significantly only in C5. NH_4_^+^-N, NO_3_^−^-N, and AK showed higher concentrations than CK in selected planted treatments (Table S2). This contrasting response may partly reflect the short 90-day restoration period, as the accumulation of SOC and TN via litter decomposition and humus formation requires longer successional timeframes to manifest [43,44]. Root-derived carbon inputs may also have contributed to the observed increase in available N by stimulating microbial mineralization, consistent with rhizosphere-priming mechanisms reported previously [45]. These treatment-specific increases in mineral N may reflect short-term, rhizosphere-associated N transformations rather than sustained changes in bulk C and N pools [45,46]. However, the spatial origin and persistence of these changes cannot be directly established from the present soil measurements. Together, these results suggest an asynchronous early recovery of substrate functions: physical and hydrological properties responded rapidly to vegetation establishment, whereas biochemical and available-nutrient responses were more treatment-specific and most total nutrient concentrations remained comparatively stable.

### 3.3. Microbial Community Reassembly During the First Growing Season

Soil microbial communities responded rapidly to vegetation establishment during the first growing season. Most planted treatments showed higher microbial richness than CK, while PERMANOVA detected significant treatment-associated differences in bacterial and fungal community composition (Figure 4e,f). However, PERMDISP indicated significant heterogeneity in within-group dispersion for fungi, but not for bacteria. Therefore, the significant fungal PERMANOVA result should be interpreted cautiously, as differences in within-group dispersion may have contributed to the observed treatment-associated variation. Given the limited number of biological replicates per treatment, these findings warrant cautious interpretation. At the taxonomic level, *Fusarium* was significantly depleted in C1, C2, and C4 relative to CK (Figure 5b). The genus *Fusarium* includes several major soil-borne plant pathogens [47,48,49], although pathogenicity of the taxa detected here was not directly assessed. Several bacterial genera containing strains previously associated with plant-beneficial functions were also enriched. For example, some *Sphingomonas* strains can produce phytohormones and promote plant growth under abiotic stress [50]. *Bradyrhizobium*, although classically associated with legume symbiosis, also includes free-living and non-symbiotic ecotypes occurring in non-leguminous environments [51,52]. Previous studies have identified *Bradyrhizobium* in alpine grassland soils of the Tibetan Plateau, including among the dominant genera in alpine meadows [53,54], and a free-living member of this genus has been characterized as oligotrophic and found to possess diverse nutrient transport and carbon metabolism-related genes [55]. Its enrichment in the absence of legume hosts is therefore consistent with the capacity of some members of this genus to persist independently of legume symbiosis. These taxonomic shifts were consistent with an early reorganization of the soil microbial community, including changes in potentially pathogenic and plant-beneficial-associated taxa. Some *Microvirga* strains have been reported as legume nodule symbionts [56], and recent work has associated this genus with plant nutrient status and microbial network structure [57]. However, the functional role of *Microvirga* in the present alpine mining system remains to be experimentally validated.

Stage-specific co-occurrence networks further showed pronounced temporal reorganization during the 90-day experimental period. The number of nodes increased from day 30 to day 90, whereas network density declined and modularity fluctuated but remained lower at day 90 than at day 30, indicating substantial topological restructuring rather than a uniform increase in network complexity (Figure 5d). Despite these changes, all stage-specific networks showed declines in relative natural connectivity under targeted node removal (Figure 5c), indicating persistent sensitivity to the simulated removal of highly connected nodes. Notably, the increase in node number was not accompanied by greater network density, which may partly explain this sensitivity. Studies of non-degraded alpine meadows have reported more complex bacterial co-occurrence networks than those in degraded or recently revegetated soils [58]. Furthermore, long-term alpine grassland restoration studies have highlighted the association between core microbial taxa, network resilience, and plant–soil system resilience [20]. Although differences in network construction methods limit direct quantitative comparisons, these findings provide a broader ecological context for the vulnerability observed in our study. Importantly, the edges in these co-occurrence networks represent statistical associations rather than experimentally verified ecological interactions, and simulated node removal constitutes an in silico perturbation of the inferred network. Therefore, the observed decline in relative natural connectivity indicates topological sensitivity but does not directly demonstrate the ecological resilience of microbial communities or their capacity to recover from actual environmental disturbances.

### 3.4. Ecological Implications of Soil–Microbial Associations During Early Revegetation

Mantel analysis (Figure 6) revealed that bacterial community structure was significantly associated with CP, NCP, and TP (*p* < 0.05). Higher CP represents a greater proportion of capillary pores, which can alter water retention, resource diffusion, and microscale habitat connectivity for soil microorganisms [26,59]. In porous soils, pore geometry and water connectivity strongly influence microbial dispersal and spatial organization [25]. Because CP and NCP represent contrasting components of soil pore-size distribution and were negatively correlated, their simultaneous associations with bacterial community structure may reflect related aspects of the soil physical environment rather than independent environmental effects. Together with the higher SWC and water-holding capacity observed in planted soils, these associations suggest a potential link between soil physical conditions and bacterial community composition. However, the Mantel analysis does not establish the direction or causality of these relationships. TP was also significantly associated with bacterial community structure. Phosphorus status has previously been linked to bacterial diversity patterns across the Qinghai–Tibet Plateau [60]. TP has recently been identified as an important environmental factor associated with bacterial community assembly in restored mining slopes on the Tibetan Plateau [61]. Longer-term vegetation succession can substantially redistribute soil P among different fractions even when total P changes little [62,63]. TP is also strongly constrained by parent material and lithology over pedogenic timescales [64,65]. Therefore, the association observed here may partly reflect background edaphic P conditions rather than solely a rapid vegetation-induced effect.

In contrast, none of the measured soil variables was significantly associated with fungal community variation (Figure 6). Previous studies likewise indicate that bacterial and fungal communities can follow distinct successional and assembly trajectories, with different sensitivities to edaphic and biotic filtering [58,66,67]. Many soil fungi establish direct associations with host plants through endophytic, mycorrhizal, or pathogenic lifestyles, and their community composition may be more closely associated with plant-related factors [68,69]. This contrast suggests that early bacterial reorganization was more closely coupled to changes in the measured substrate environment, whereas fungal variation may have depended on additional plant-associated, biotic, or unmeasured environmental factors.

Because the microbial analyses were based on amplicon sequencing, the ecological functions inferred for individual taxa cannot be directly verified from the present data [70]. Future shotgun metagenomics and targeted cultivation or functional assays could help determine the metabolic roles of candidate taxa identified during early restoration [71].

## 4. Materials and Methods

### 4.1. Study Site and Experimental Design

The ecological restoration experiment was conducted at the Kaerqing construction sand and gravel mine in Tangga Township, Dazi District, Lhasa City (29°50′48″ N, 91°33′12″ E) (Figure S1). The study area features an alpine cool semi-arid climate, characterized by small annual temperature variations, large daily temperature fluctuations, and strong solar radiation. Annual sunshine hours are approximately 3065, with a frost-free period of about 130 days. The mean annual daily maximum and minimum temperatures are approximately 17.0 °C and 1.0 °C, respectively. Annual average precipitation is roughly 450 mm, and vegetation growth primarily relies on natural precipitation. The site is situated at an altitude of 3500–4000 m, belonging to a typical alpine meadow ecosystem. The dominant herbaceous species is *E. nutans*, accompanied by other native perennial grasses such as *Festuca* spp., *Agropyron* spp., and *Poa* spp.

Three pioneer grass species, *E. nutans*, *A. cristatum*, and *F. sinensis*, were selected for the revegetation experiment. Eight treatments were established, comprising three monocultures, four mixed-sowing combinations, and a bare soil control: C1 (*E. nutans*), C2 (*A. cristatum*), C3 (*F. sinensis*), C4 (*E. nutans* + *A. cristatum*, 1:1), C5 (*E. nutans* + *F. sinensis*, 1:1), C6 (*A. cristatum* + *F. sinensis*, 1:1), C7 (*E. nutans* + *A. cristatum* + *F. sinensis*, 1:1:1), and CK (bare control). To standardize the sowing rate across planting treatments, a total of 6000 seeds·m^−2^ was used for all planted treatments, with constituent species in mixed-sowing treatments distributed in equal seed proportions. As the seeds were purchased locally and seed-quality testing facilities were not available at the experimental site, seed purity and germination rates were not assessed before sowing. Accordingly, sowing rates were standardized based on the number of seeds sown, without adjustment for seed purity or germination rates. In June 2025, gravel was cleared, and 3 m × 3 m plots were established, separated by partition boards. The eight treatments were randomly assigned to the plots, with three replicate plots per treatment. Seeds were soaked in a 3% H_2_O_2_ solution for 30 min for surface disinfection and rinsed thoroughly with tap water. Unless otherwise specified, all chemical reagents used in this study were purchased from Shanghai Macklin Biochemical Technology Co., Ltd. (Shanghai, China) through a local distributor. After plowing and sowing, the plots were watered promptly. Manual watering was applied only during the first week, with no further human intervention thereafter.

### 4.2. Experimental Recording and Sampling

The average plant height in each plot was recorded in July, August, and September 2025 (at 30-day intervals). Corresponding topsoil samples (0–20 cm) were collected simultaneously from the same 24 experimental plots at each sampling time using a five-point sampling method. The five subsamples collected within each plot were combined into one composite soil sample, with each plot representing an independent biological replicate. Before sowing (day 0), soil samples were collected across the experimental plot area and from adjacent undisturbed reference soil to characterize their initial physical, chemical, and biochemical properties (Table S1). The day-0 samples represented the overall baseline conditions of the experimental area. Treatment effects at day 90 were evaluated relative to the contemporaneous CK. In this study, early recovery refers to vegetation establishment and improvements in selected soil physical and hydrological properties relative to CK, alongside microbial community reassembly, rather than a return to undisturbed reference-soil conditions.

Plants were harvested after 90 days. Plant samples collected within each plot were pooled and divided into two parts: one part of the leaves was immediately frozen at −80 °C in an ultra-low-temperature freezer (DW-HL538, Zhongke Meiling Cryogenics Co., Ltd., Hefei, China) for subsequent physiological index determinations, and the remainder was used for biomass measurement. Soil samples were initially passed through a 10-mm sieve to remove gravel. A subsample was stored at −80 °C for DNA extraction, while the remaining soil was transported to the laboratory for further physicochemical analyses.

### 4.3. Soil Sample Analysis

Soil pH and electrical conductivity (EC) were measured using a SevenExcellence S470-K pH/conductivity meter (Mettler Toledo Technology (China) Co., Ltd., Shanghai, China) at a soil-to-water ratio of 1:2.5 (*w*/*v*). Cation exchange capacity (CEC) was determined via the hexamminecobalt trichloride spectrophotometric method [72], with absorbance measured using an SP-Max 2300A2 microplate reader (Shanghai Flash Spectrum Biological Technology Co., Ltd., Shanghai, China). Soil water content (SWC), capillary porosity (CP), non-capillary porosity (NCP), bulk density, saturated moisture content, capillary water-holding capacity, and field capacity were evaluated using the intact soil core method. The analytical methods for available potassium (AK), ammonium nitrogen (NH_4_^+^-N), nitrate nitrogen (NO_3_^−^-N), total nitrogen (TN), total phosphorus (TP), available phosphorus (AP), total potassium (TK), soil organic carbon (SOC), and enzyme activities are described in Text S1.

### 4.4. Plant Sample Analysis

Harvested plants were washed with deionized water and separated into root and aboveground parts. The pre-frozen leaf samples (stored at −80 °C) were used to determine photosynthetic pigment contents, malondialdehyde (MDA) content, and antioxidant enzyme activities on a fresh weight (FW) basis. The remaining plant materials were placed in a forced-air drying oven (DHG-9000 series, Shanghai Yiheng Scientific Instrument Co., Ltd., Shanghai, China) at 105 °C for 30 min and then dried at 75 °C to a constant weight for biomass determination. Photosynthetic pigments, including chlorophyll a (Chl a), chlorophyll b (Chl b), and carotenoids (Car), were extracted using 80% acetone, and their absorbance was measured using the same SP-Max 2300A2 microplate reader. MDA was assessed using the thiobarbituric acid (TBA) reaction method. The activities of peroxidase (POD), superoxide dismutase (SOD), and catalase (CAT) were quantified using commercial assay kits (Nanjing MoFan Biotechnology, Nanjing, China). Absorbance measurements for the MDA and antioxidant enzyme assays were also performed using this microplate reader.

### 4.5. Microbial Analysis

Total genomic DNA was extracted from the soil samples. The amplicons of the bacterial 16S rRNA gene (V4 region) and fungal internal transcribed spacer 1 (ITS1) region were sequenced on the MGISEQ-2000RS sequencer (MGI Tech Co., Ltd., Shenzhen, China). Detailed procedures for DNA extraction, PCR amplification, sequencing, and bioinformatic processing are provided in Text S2.

At day 90, principal coordinate analysis (PCoA) based on Bray–Curtis dissimilarities was used to visualize bacterial and fungal community composition. To evaluate treatment-associated differences, PERMANOVA was performed separately for bacterial and fungal communities using Bray–Curtis dissimilarity matrices calculated from the ASV abundance tables. The analysis included 24 samples for each microbial group, representing eight treatments with three independent biological replicates per treatment. Statistical significance was assessed using 9999 permutations of sample group labels. To determine whether the PERMANOVA results could be influenced by differences in within-group dispersion, homogeneity of multivariate dispersion was evaluated using PERMDISP. Distances from individual samples to their respective group spatial medians were calculated in PCoA space, and significance was assessed using 9999 permutations of model residuals. At day 90, differential abundance analysis was performed at the genus level using DESeq2 package (version 1.22.1) in R (version 3.5.1) on a combined abundance matrix comprising bacterial 16S rRNA and fungal ITS taxa derived from the cleaned ASV abundance data. The 21 samples from the seven planted treatments (C1–C7; three biological replicates per treatment) were assigned to a single planted group (C), which was compared with the three bare-soil control samples (CK). Abundance values were rounded to integers before analysis, and low-abundance taxa were filtered by retaining those with counts per million (CPM) greater than 1 in at least two samples. DESeq2 analysis was performed using a two-group design (design = ~conditions), with size factors estimated using the ratio method (type = “ratio”) and differential abundance assessed using the Wald test. *p*-values were adjusted for multiple testing using the Benjamini–Hochberg procedure. The contrast was defined as C versus CK, and genera with |log_2_FC| ≥ 1 and an FDR-adjusted *p*-value (padj) < 0.05 were considered differentially abundant. Positive and negative log_2_FC values indicated enrichment and depletion, respectively, in the pooled planted group relative to CK. PCoA and DESeq2 analyses were performed using the Metware Cloud, a free online platform for data analysis (Metware Biotechnology Co., Ltd., Wuhan, China; https://cloud.metware.cn, accessed on 20 August 2026). PERMANOVA results were independently recalculated, and PERMDISP analyses were conducted using permutation analyses implemented in Python (version 3.13.5) on the exported ASV abundance data.

To explore microbial interactions, co-occurrence networks of microbiomes across the three experimental stages (30, 60, and 90 days) were constructed by integrating all 21 samples (7 planted treatments with 3 biological replicates each) per time point. Spearman’s rank correlations were calculated among microbial genera, retaining only strong associations (|r| > 0.8, Benjamini–Hochberg-adjusted *p* < 0.05). The relatively stringent correlation threshold was selected to focus on strong associations and limit the inclusion of weak correlations in the inferred networks. Network construction was performed using the “psych (version 2.6.5)” and “microeco (version 2.3.0)” packages in R (version 4.5.0). The topological structures of the networks were visualized using the Gephi software (version 0.10.1). The topological robustness of the inferred co-occurrence networks was evaluated using relative natural connectivity under simulated random and targeted node removal (0–80%, at 2% intervals). Random removal was repeated 100 times, whereas targeted removal followed a fixed descending order of node degrees calculated from the original network. Natural connectivity was calculated from the eigenvalues of the unweighted adjacency matrix and normalized to that of the intact network. A faster decline indicated greater topological vulnerability to simulated node loss rather than directly demonstrating lower ecological resilience.

The raw bacterial 16S rRNA gene and fungal ITS amplicon sequencing data generated in this study have been deposited in the NCBI Sequence Read Archive (SRA) under BioProject accession PRJNA1537329.

### 4.6. Data Processing and Statistical Analysis

Means and standard deviations were calculated using Microsoft Office Excel 2024. Data normality and homogeneity of variance were verified via Shapiro–Wilk and Levene’s tests, with log-transformations applied when necessary to meet normal distribution assumptions. Statistical comparisons among the eight treatments were conducted using data collected at day 90. The experimental plot was considered the independent biological replicate (*n* = 3 per treatment). Treatment differences were evaluated using one-way ANOVA followed by Tukey’s HSD test (*p* < 0.05) in GraphPad Prism 10.1.2. Temporal patterns in mean plant height, soil pH, and soil water content at days 30, 60, and 90 were presented descriptively, without formal hypothesis testing for time effects or treatment-by-time interactions. Because repeated observations were obtained from the same experimental plots, measurements from different sampling times were not pooled as independent replicates for statistical comparisons.

Plots were generated using Origin 2024 (OriginLab, Northampton, MA, USA). To identify soil physicochemical factors associated with early microbial community reorganization, Mantel tests were performed using the mantel_test() function in the ggcor package (version 0.9.8.1), which uses the vegan package (version 2.7-6) for Mantel testing, in R. Bray–Curtis dissimilarities were calculated for microbial community abundance data, whereas Euclidean distances were calculated for soil environmental variables. Pearson correlation coefficients were computed in R to evaluate the pairwise relationships among the 18 soil microhabitat indicators. The integrated results were visualized as a combined correlation heatmap.

## 5. Conclusions

A single growing season (90 days) of pioneer revegetation was sufficient to initiate measurable plant–soil–microbial recovery in the alpine mining substrate. Soil physical and hydrological properties showed the most consistent early responses, with pronounced changes in pore structure, soil moisture, and water-holding capacity, whereas biochemical and nutrient responses were less consistent and more treatment-dependent. This pattern indicates asynchronous recovery of substrate functions, with physical and hydrological properties showing clearer early responses than bulk nutrient status. Meanwhile, bacterial and fungal communities underwent significant reorganization, and stage-specific co-occurrence networks showed clear temporal restructuring. However, the inferred networks remained sensitive to simulated targeted node removal. Bacterial community variation was significantly associated with CP, NCP, and TP, suggesting a potential relationship between soil physicochemical conditions and early bacterial community reassembly, although the direction and causality of these associations remain unresolved. Overall, 90-day revegetation initiated habitat reconstruction rather than completing ecosystem recovery, and this short timescale is insufficient to determine the long-term superiority of monoculture or mixed-sowing configurations.

## Data Availability

The raw bacterial 16S rRNA gene and fungal ITS amplicon sequencing data generated in this study are publicly available in the NCBI Sequence Read Archive (SRA) under BioProject accession PRJNA1537329.

## Supplementary Materials

The following supporting information can be downloaded at: https://www.mdpi.com/article/10.3390/plants15203173/s1, Text S1: Determination of substrate chemical properties (soil enzyme assay methods based on [73]); Text S2: Details of DNA extraction, PCR amplification, sequencing, and bioinformatic processing; Table S1: Initial physical, hydrological, chemical, and biochemical properties of the disturbed mining substrate and adjacent undisturbed reference soil at day 0; Table S2: Soil nutrient characteristics under different treatments at day 90; Figure S1: Location of the study site and experimental design of the planting treatments; Figure S2: Photosynthetic pigment contents (Chl a, Chl b, and Car) of individual plant species across different treatments; Figure S3: Soil bulk density under different treatments at day 90.

## Abbreviations

The following abbreviations are used in this manuscript:

DNA	Deoxyribonucleic acid
EC	Electrical conductivity
CEC	Cation exchange capacity
SWC	Soil water content
CP	Capillary porosity
NCP	Non-capillary porosity
AK	Available potassium
NH_4_^+^-N	Ammonium nitrogen
NO_3_^−^-N	Nitrate nitrogen
TN	Total nitrogen
TP	Total phosphorus
AP	Available phosphorus
TK	Total potassium
SOC	Soil organic carbon
MDA	Malondialdehyde
FW	Fresh weight
Chl a	Chlorophyll a
Chl b	Chlorophyll b
Car	Carotenoids
TBA	Thiobarbituric acid
POD	Peroxidase
SOD	Superoxide dismutase
CAT	Catalase
16S rRNA	16S ribosomal RNA
ITS1	Internal transcribed spacer 1
PCR	Polymerase chain reaction
PCoA	Principal coordinate analysis
PERMANOVA	Permutational multivariate analysis of variance
FDR	False discovery rate
ASVs	Amplicon sequence variants
ANOVA	Analysis of variance
HSD	Honestly significant difference
SUC	Sucrase
ALP	Alkaline phosphatase
URE	Urease
ROS	Reactive oxygen species
