# Supplementary material for: Early Revegetation Initiates Soil Physical Habitat Reconstruction and Microbial Community Reassembly Within the First Growing Season in an Alpine Mining Substrate
Source: Plants (Basel). 2026 Oct 16;15(20):3173. doi: 10.3390/plants15203173 (PMC13708970; doi:10.3390/plants15203173)
Supplement: Supplementary file 1 [file plants-15-03173-s001.zip › plants-4633472-supplementary.pdf]

Supplementary Material for

Early revegetation initiates soil physical habitat reconstruction and microbial community reassembly within the first growing season in an alpine mining substrate

Shuhan Zhang[1], Bo Cai[1], Xiangqian Zhao[1], Jing Xiang[1], Xihui Sun[1], Lixin Niu[1], Xianzhi Wang[2]*, Yanlong Zhang[1]*

1. College of Landscape Architecture & Arts, Northwest A&F University, Yangling, 712100, China

2. China MCC5 Group Corp. Ltd., Chengdu, 610063, China

* Corresponding authors: Xianzhi Wang (115024967@163.com), Yanlong Zhang (zhangyanlong@nwafu.edu.cn).

This supplementary material contains a 6-page document, including 2 texts, 2 tables and 3 figures. Notes: C1, *E. nutans*; C2, *A. cristatum*; C3, *F. sinensis*; C4, *E. nutans* + *A. cristatum*, 1:1; C5, *E. nutans* + *F. sinensis*, 1:1; C6, *A. cristatum* + *F. sinensis*, 1:1; C7, *E. nutans* + *A. cristatum* + *F. sinensis*, 1:1:1; and CK, bare control.

Text S1

Determination of substrate chemical properties

Available potassium (AK) was extracted with ammonium acetate and determined using an M410 flame photometer (Sherwood Scientific Ltd., Cambridge, UK). Ammonium nitrogen (NH_4^+-N) and nitrate nitrogen (NO_3^--N) were extracted with KCl and analyzed using a SAN++® Classic continuous flow analyzer (Skalar Analytical B.V., Breda, The Netherlands). Available phosphorus (AP) was extracted with sodium bicarbonate and determined via the molybdenum blue method. Total nitrogen (TN), total phosphorus (TP), and total potassium (TK) were digested with H_2SO_4-H_2O_2; subsequently, TN and TP were analyzed using the SAN++® analyzer, while TK was determined using the same flame photometer. Soil organic carbon (SOC) was quantified by the potassium dichromate oxidation-spectrophotometric method. For soil enzyme activities, urease was measured by the phenol-sodium hypochlorite colorimetric method, sucrase by the 3,5-dinitrosalicylic acid colorimetric method, catalase by potassium permanganate titration, and alkaline phosphatase by the disodium phenyl phosphate colorimetric method [73].

Text S2

Details of DNA extraction, PCR amplification, sequencing, and bioinformatic processing

Total genomic DNA was extracted from soil samples using the CretMag™ Power Soil DNA Kit (CretBiotech Co., Ltd., Suzhou, China) according to the manufacturer's instructions. DNA quality was assessed by 1% agarose gel electrophoresis, and DNA concentration and purity were determined using a NanoDrop 2000 UV-vis spectrophotometer (Thermo Scientific, Wilmington, DE, USA). The extracted genomic DNA was used as the template for PCR amplification with ES Taq MasterMix (Dye) (Jiangsu Cowin Biotech Co., Ltd., Taizhou, China), and PCR reactions were performed in triplicate for each sample. The V4 region of the bacterial 16S rRNA gene was amplified using primers 515F and 806R, whereas the fungal internal transcribed spacer 1 (ITS1) region was amplified using primers ITS1F and ITS2. PCR products from the same sample were pooled and examined by 2% agarose gel electrophoresis. Qualified PCR products were purified using the AxyPrep DNA Gel Extraction Kit (Axygen Biosciences, Union City, CA, USA), verified again by 2% agarose gel electrophoresis, and quantified using a Quantus™ Fluorometer (Promega Corporation, Madison, WI, USA). Sequencing libraries were constructed using the VAHTS® Universal Plus DNA Library Prep Kit for MGI V2 (Vazyme Biotech Co., Ltd., Nanjing, China) and sequenced on an MGISEQ-2000RS high-throughput sequencer (MGI Tech Co., Ltd., Shenzhen, China).

Paired-end reads were assigned to samples according to their unique barcode sequences, followed by removal of barcode and primer sequences. Quality filtering was performed using fastp (version 0.22.0) to obtain high-quality clean reads. Paired-end reads were subsequently merged using FLASH (version 1.2.11). Chimeric sequences were identified using the UCHIME algorithm implemented in VSEARCH (version 2.22.1) by

comparison against the SILVA database (release 138.1) for bacterial sequences and the UNITE database (version 9.0) for fungal sequences and were subsequently removed to obtain effective tags. Amplicon sequence variants (ASVs) were generated using Deblur (version 1.1.1). Taxonomic annotation of bacterial ASVs was performed against the SILVA database using the Mothur algorithm (version 1.48), whereas fungal ASVs were annotated against the UNITE database using the Mothur algorithm. Multiple sequence alignment was performed using MAFFT (version 7.520) to infer phylogenetic relationships among representative sequences. The ASV abundance table was normalized to the sequencing depth of the sample with the lowest number of sequences, and subsequent alpha- and beta-diversity analyses were performed using the normalized dataset.

Table S1 Initial physical, hydrological, chemical, and biochemical properties of the disturbed mining substrate and adjacent undisturbed reference soil at day 0. Results are means ± SD (*n* = 3).

Index	Disturbed mining substrate	Adjacent undisturbed reference soil
SWC/%	2.61 ± 0.38	8.91 ± 4.10
Saturated water content/g·kg^{-1}	158.07 ± 28.49	288.60 ± 43.96
Capillary water-holding capacity/g·kg^{-1}	134.02 ± 25.77	264.72 ± 37.50
Field capacity/g·kg^{-1}	102.70 ± 25.36	193.96 ± 39.40
Bulk density/g·cm^{-3}	1.64 ± 0.14	1.45 ± 0.13
NCP/%	3.91 ± 1.25	3.40 ± 0.88
CP/%	21.76 ± 2.67	38.12 ± 3.07
Total porosity/%	25.66 ± 2.75	41.52 ± 3.47
pH	8.38 ± 0.03	6.66 ± 0.03
EC/μS·cm^{-1}	98.57 ± 3.14	39.40 ± 1.31
CEC/cmol(+)·kg^{-1}	0.97 ± 0.61	3.45 ± 0.29
SOC/g·kg^{-1}	3.02 ± 0.14	11.07 ± 0.79
TN/g·kg^{-1}	0.43 ± 0.02	0.84 ± 0.03
TP/g·kg^{-1}	1.08 ± 0.06	1.11 ± 0.02
TK/g·kg^{-1}	3.77 ± 0.31	4.67 ± 0.00
AP/mg·kg^{-1}	3.53 ± 0.30	7.05 ± 0.46
AK/mg·kg^{-1}	47.34 ± 0.00	135.81 ± 8.06
NH_4^+-N/mg·kg-1	1.36 ± 0.04	2.37 ± 0.25
NO_3^--N/mg·kg-1	3.42 ± 0.57	11.46 ± 1.40
SUC/mg·g^{-1}·24h^{-1}	6.10 ± 0.96	154.33 ± 2.87
URE/mg·g^{-1}·24h^{-1}	0.08 ± 0.01	0.54 ± 0.02
CAT/mL·g^{-1}·20min^{-1}	0.65 ± 0.05	0.98 ± 0.03
ALP/mg·g^{-1}·24h^{-1}	0.19 ± 0.14	0.97 ± 0.36

Table S2 Soil nutrient characteristics under different treatments at day 90

Results are means ± SD (*n*=3). Different lowercase letters indicate significant differences among treatments according to Tukey's HSD test ($p < 0.05$).

Index	SOC (g·kg^{-1})	TN (g·kg^{-1})	TP (g·kg^{-1})	TK (g·kg^{-1})	AP (mg·kg^{-1})	AK (mg·kg^{-1})	NH_4^+-N (mg·kg^{-1})	NO_3^--N (mg·kg^{-1})
C1	4.42 ± 0.98a	0.5 ± 0.04ab	1.72 ± 0.1ab	4.13 ± 0.54b	5.56 ± 0.79ab	75.28 ± 0b	1.79 ± 0.03a	6.39 ± 0.17b
C2	2.28 ± 0.15b	0.41 ± 0.01b	1.46 ± 0.11b	4.86 ± 0.83b	4.45 ± 0.15b	47.34 ± 0d	0.67 ± 0.04c	1.29 ± 0.34cd
C3	3.06 ± 0.39ab	0.51 ± 0.07a	1.35 ± 0.06b	4.49 ± 0.31b	4.07 ± 0.56b	89.24 ± 0a	0.66 ± 0.05c	15.81 ± 0.2a
C4	3.75 ± 1.26ab	0.41 ± 0.02b	1.57 ± 0.2b	5.22 ± 0.94b	4.11 ± 0.2b	47.34 ± 0d	0.64 ± 0.05c	0.71 ± 0.13d
C5	2.81 ± 0.55ab	0.42 ± 0.03b	1.27 ± 0.05bc	7.21 ± 0.31a	5.71 ± 1.7b	52 ± 8.06cd	1.09 ± 0.03b	1.95 ± 0.13cd
C6	3.74 ± 0.67ab	0.45 ± 0.03ab	1.57 ± 0.2b	6.67 ± 0.31ab	5.46 ± 0.43ab	61.31 ± 0c	1.06 ± 0.02bc	2.82 ± 0.07c
C7	2.68 ± 0.27ab	0.37 ± 0.02b	1.94 ± 0.05a	3.95 ± 0.31b	6.29 ± 0.74a	56.65 ± 8.06cd	1.01 ± 0.06bc	0.97 ± 0.69cd
CK	2.75 ± 0.25ab	0.43 ± 0.01ab	0.98 ± 0.04c	4.13 ± 0b	4.84 ± 0.56ab	47.34 ± 0d	0.74 ± 0.29c	1.71 ± 1.48d

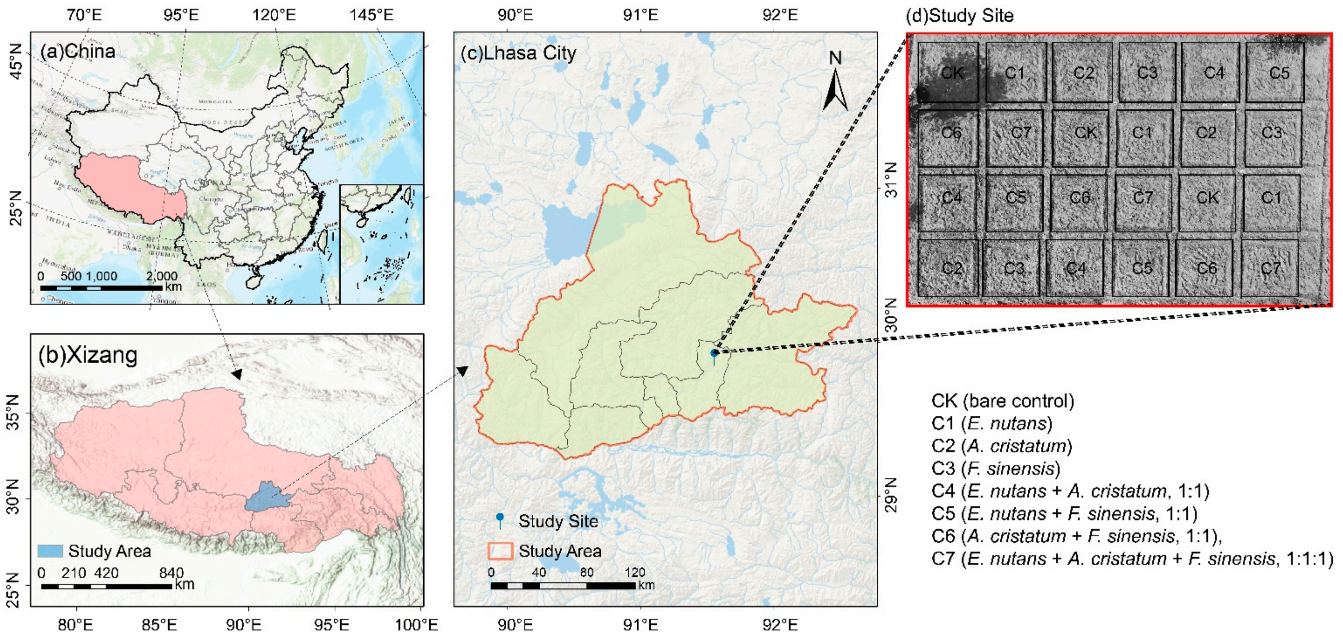

Fig. S1. Location of the study site and experimental design of the planting treatments.

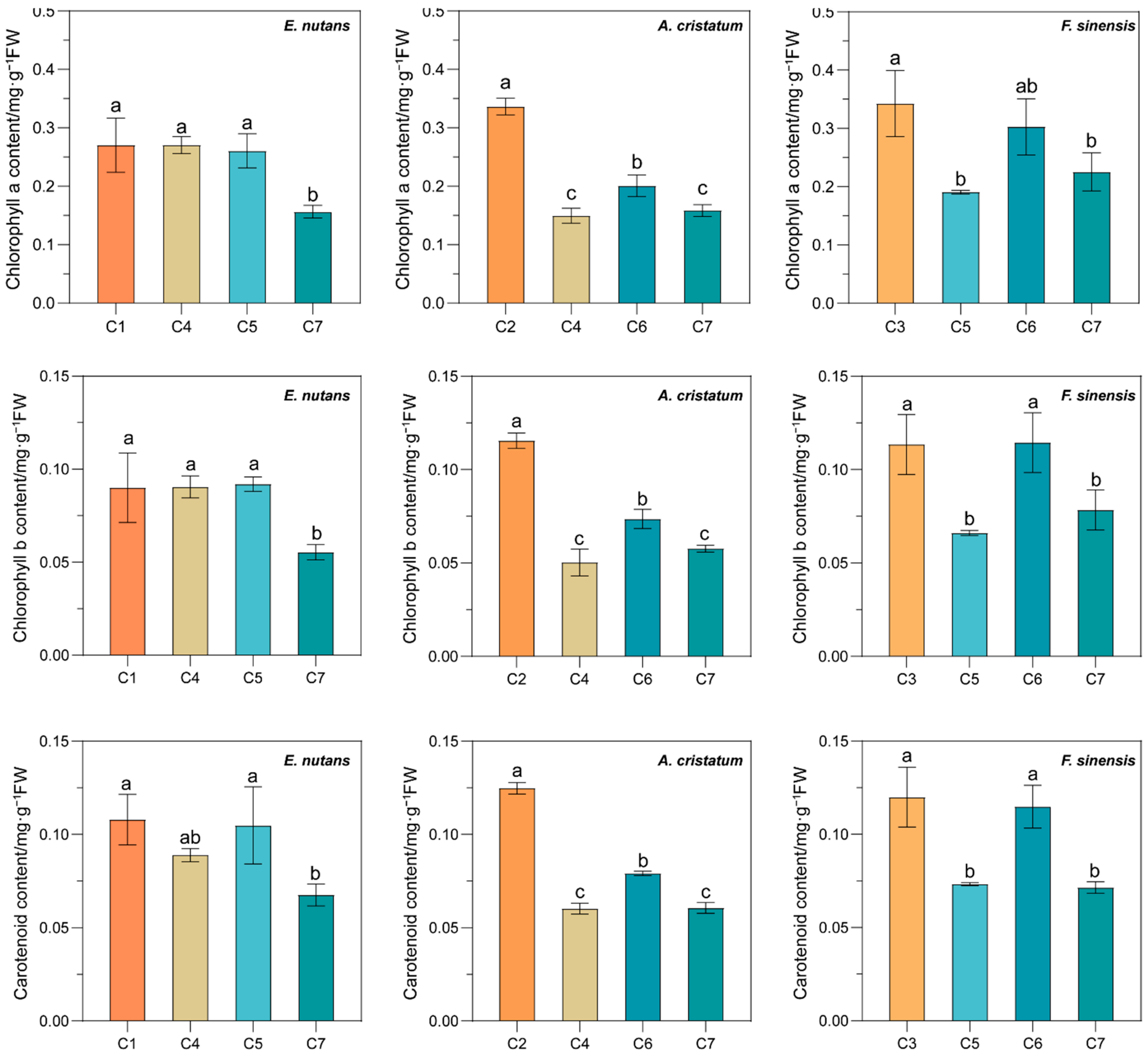

Fig. S2. Photosynthetic pigment contents (Chl a, Chl b, and Car) of individual plant species across different treatments.

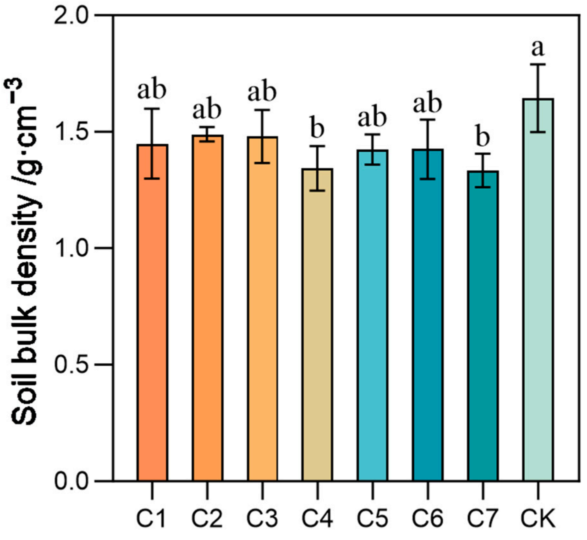

Fig. S3. Soil bulk density under different treatments at day 90.

References

73. Jiang, X.; Zhong, X.; Yu, G.; Zhang, X.; Liu, J. Different Effects of Taproot and Fibrous Root Crops on Pore Structure and Microbial Network in Reclaimed Soil. Sci. Total Environ. 2023, 901, 165996, doi:10.1016/j.scitotenv.2023.165996.
